# Cortical cholinergic pathway integrity is reduced in Parkinson’s disease dementia

**DOI:** 10.1002/alz.091130

**Published:** 2025-01-09

**Authors:** Nicola M Slater, Daniel J Myall, Kyla‐Louise Horne, Tim J Anderson, Campbell Le Heron, Tracy R Melzer, John C Dalrymple‐Alford

**Affiliations:** ^1^ University of Canterbury, Christchurch New Zealand; ^2^ New Zealand Brain Research Institute, Christchurch New Zealand; ^3^ University of Otago, Christchurch New Zealand; ^4^ Christchurch Hospital, Christchurch, Canterbury New Zealand

## Abstract

**Background:**

Normal cognitive function is supported by the neuromodulatory mechanisms of the cholinergic system. In Parkinson’s disease (PD), histological and molecular imaging evidence suggests disruption to cholinergic circuitry is associated with progression to Parkinson’s disease dementia (PDD). The primary source of cortical cholinergic input is the Ch4 region of the basal forebrain. Reduction in cholinergic projection axon integrity may precede Ch4 neuronal decline, so we examined differences in the integrity of medial and lateral Ch4‐to‐cortex pathways from three subregions of Ch4.

**Method:**

Structural and diffusion‐weighted MRI and neuropsychological assessment was acquired from a convenience sample of 108 PD patients, 60 with normal cognition, 37 with mild cognitive impairment, 12 PDD, and 41 control participants. Streamlines connecting the anterior‐lateral, anterior‐intermediate, and posterior Ch4 subregions to the cortex were grouped according to medial and lateral cortical cholinergic pathway definitions by Selden et al. [1]. Integrity was assessed using diffusion tensor, NODDI, and fixel‐based models.

**Results:**

Accounting for age and sex, Bayesian mixed‐effects models indicated moderate to strong evidence of increased free water in PDD in all pathways, especially in connections with anterior‐intermediate and posterior Ch4 subregions (Cohen’s d (Control–PDD) = 0.47). Across connections with all subregions there was moderate to strong evidence of increased mean diffusivity (Cohen’s d (Control–PDD) = 0.76) and reduced fibre density (Cohen’s d (Control–PDD) = 1.24) in PDD. In PD‐MCI, moderate evidence of increased mean diffusivity was observed (Cohen’s d (Control–PD‐MCI)= 0.64), but no evidence for alterations in other metrics.

**Conclusion:**

Our results reveal evidence of reduced integrity in the major cortical cholinergic pathways in PD‐MCI, and more extensive evidence of reduced integrity in PDD. While these findings support an association of cholinergic circuity disruption in PD‐related cognitive impairment, they contrast with a commonly held view that losses in cholinergic projection integrity may explain, and potentially precede, cognitive impairment in PD. Ch4 projection pathway integrity may be more pertinent to future cognitive decline than current cognitive status. Longitudinal investigation will examine how Ch4 projection pathway integrity relates to the progression of cognitive decline in PD.

**Reference**:

[1] Selden, et al. Brain 121, 2249–2257 (1998).

